# An Observational Study of 147 Psoriasis Patients: Overweightness and Obesity as a Significant Clinical Factors Correlated with Psoriasis

**DOI:** 10.3390/medicina59112006

**Published:** 2023-11-15

**Authors:** Anna Czarnecka, Monika Zabłotna, Dorota Purzycka-Bohdan, Roman J. Nowicki, Aneta Szczerkowska-Dobosz

**Affiliations:** Department of Dermatology, Venereology and Allergology, Medical University of Gdansk, 80-214 Gdansk, Poland; monika.zablotna@gumed.edu.pl (M.Z.); dorota.purzycka-bohdan@gumed.edu.pl (D.P.-B.); rnowicki@gumed.edu.pl (R.J.N.); aneta.szczerkowska-dobosz@gumed.edu.pl (A.S.-D.)

**Keywords:** obesity, psoriasis, BMI, PASI, BSA

## Abstract

*Background and Objectives*: Psoriasis is a common, chronic, and immune-mediated inflammatory skin disease recognized to lead to a wide range of comorbid disorders, mainly obesity. The study aimed to evaluate the problem of overweightness and obesity among psoriasis patients in the context of their prevalence and influence on the disease course. *Materials and Methods*: The study group encompassed 147 adult patients with plaque psoriasis. *Results*: The prevalences of overweightness (39.46%) and obesity (37.41%) demonstrated in the study showed the strong predisposition of psoriatic patients for abnormal body mass. The vast majority (77%) of subjects with psoriatic arthritis were overweight or obese. The results of the correlation analysis revealed the significant impacts of overweightness and obesity, as defined by the BMI index, on modifying the severity of psoriasis (as assessed by the PASI with a correlation coefficient of R = 0.23, *p* = 0.016; and BSA values with a correlation coefficient of R = 0.21, *p* = 0.023), particularly in contrast to patients with a normal body mass. *Conclusions*: Overweightness and obesity constitute a major health burden among psoriatic patients, influencing the disease course and severity. Enhanced understanding of the phenomenon may directly translate into improving disease management and overall patient care.

## 1. Introduction

Psoriasis is a common, chronic, and immune-mediated inflammatory skin disease characterized by the formation of erythematous plaques covered with silvery-white scales. The worldwide psoriasis prevalence varies from 1–3% around the globe, with variations in incidence across different regions and ethnicities [1]. Psoriasis etiology derives from a complex interplay between manifold factors, including immunological pathways, genetic predisposition, and environmental aspects (chiefly emotional stress, smoking habits, lifestyle, diet, physical activity, and infections) [2]. Moreover, in accordance with the newest research on psoriasis pathogenesis, it is associated with a state of chronic systemic inflammation, thus identifying it as a systemic disorder. Furthermore, it is recognized to lead to a wide range of comorbid disorders, mainly obesity, hypertension, diabetes, metabolic syndrome, cardiovascular disease (CVD), and depression [3,4].

Epidemiological studies have consistently demonstrated a strong and bidirectional relationship between psoriasis and obesity, with each condition exacerbating the severity and progression of the other [5,6,7]. The fundamental pathomechanism underlying this association is the chronic state of low-grade systemic inflammation, which significantly contributes to the development and progression of both conditions. Systemic inflammation and consecutively exacerbated activation of keratinocytes promote the formation of psoriasis skin lesions by activating the immune cascade involving dendritic cells, T-helper lymphocytes (chiefly Th1, Th17, and Th22), and proinflammatory molecules (mainly IL-1, IL-6, TNF-α, IL-12, and IL-23). Furthermore, excessive secretions of adipokines and hypoxia, especially leptin and adiponectin, are a crucial aspect of the pathophysiological link between psoriasis and obesity. Obesity promotes the additional release of proinflammatory state reagents in the systemic circulation that further exacerbates epithelial and keratinocytes destruction, which contributes to a self-generating process. What is more, emerging research suggests that alterations in gut microbiota composition, known as dysbiosis, may contribute to both psoriasis and obesity. Imbalance and dysfunction of gut microorganisms were reported in both conditions, which promoted the increased permeability of the intestinal barrier. The phenomenon is associated with the translocation of proinflammatory metabolites and immune system modulators into the systemic circulation, which add up to the underlying chronic state of systemic inflammation [5].

Obesity emerges to play a key role in the development of psoriasis and associated comorbidities [8,9]; therefore, it constitutes a substantial burden to healthcare. It can impact the intensity and early onset of the condition, diminish the quality of life for affected individuals, modify the effectiveness of psoriasis treatments, and contribute to increased morbidity through a decreased lifespan attributed to cardiovascular events [10,11,12]. Therefore, investigating the prevalence and impact of comorbidities associated with psoriasis is imperative in order to provide patients with a comprehensive and holistic approach to their care. Focusing scientific research on the molecular pathogenesis of shared inflammatory pathways of psoriasis comorbid diseases may provide, in the future, new therapeutic targets centered on key molecules, which provide a multidirectional course of action. It would be a breakthrough in the context of a more diverse array of drug administration methods and subsequently increase patients’ treatment adherence. Additionally, it holds the potential to substantially diminish the risk of drug-to-drug interactions (particularly among the subpopulation of geriatric patients), as well as enhance the quality of life by simultaneously addressing multiple comorbidities and more pharmacoeconomic treatments. Such an approach aligns with the principles of patient-centered care and promotes a more comprehensive understanding of the complex interplay between psoriasis and other health conditions [13].

Moreover, the link between psoriasis and obesity is also significantly influenced by environmental and lifestyle factors, notably including dietary choices and levels of physical activity, which serve as co-factors in this association. There is growing evidence that nutrition may modulate the systemic state of inflammation with components of antioxidant and anti-inflammatory actions, such as high intakes of monosaturated and polyunsaturated fatty acids, dietary fibers, omega-3 acids, certain polyphenols, and vitamins A, E, and C, as well as oligoelements. On the other hand, the overabundance of saturated fats and unsaturated fatty acids from the *n*-6 family may trigger an increase in concentration of pro-inflammatory interleukins and contribute to the development of systemic inflammation. A growing number of studies also provide evidence for the influence of nutrients on the molecular level and epigenetic alterations from the modulation of miRNAs, which underscores the complex nature of psoriasis [14]. Research has indicated that adopting specific dietary approaches can be beneficial in both complementing psoriasis treatment and minimizing its exacerbation. These include low-energy, vegan, and vegetarian diets, as well as weight loss programs incorporating modified dietary strategies. Additionally, gluten-free, Mediterranean, and very-low-calorie diets devoid of carbohydrates have shown promise in supporting individuals with psoriasis [15]. Furthermore, the results of the meta-analysis studies demonstrated a significant association between obesity and vitamin D levels, with an increased prevalence of vitamin D deficiency among obese and overweight populations (defined by BMI index) in comparison to lean subjects, irrespective of age or ethnicity [16]. Thus, it is hypothesized that obese psoriatic patients are more prone to the skin exacerbation due to vitamin D depletion, which can result from both improper nutrition intake and overabundance of adipose tissue [17]. Finally, regular physical activity constitutes an important confounder in psoriasis development and management by regulating energy homeostasis and adipose tissue abundance, which regulate anti-inflammatory cell adaptation and the enhancement of immune system functions. However, recently published data provide evidence for the growing phenomenon of reduced physical activity among psoriatic patients [14].

Therefore, the study aimed to evaluate the problem of overweightness and obesity among psoriasis patients in the context of their prevalence and influence on the disease course.

## 2. Materials and Methods

### 2.1. Materials

An observational study was conducted between 2021 and 2023 at the Department of Dermatology, Venereology, and Allergology of the Medical University of Gdańsk, along with the Dermatological Outpatient Clinic (northern Poland region). The study group encompassed 147 adult patients with chronic plaque psoriasis (based on either clinical or histopathological diagnosis). The research protocol was approved by the Independent Bioethics Committee for Scientific Research (NKBBN/2/2021).

### 2.2. Methods

Each participant underwent demographic and clinical data collection, as well as medical examination, which focused on psoriasis onset, clinical manifestations of psoriasis and nail involvement, lifestyle factors (diet and smoking), presence of psoriatic arthritis (diagnosis based on rheumatological consultation), the severity of psoriasis (defined by the PASI—Psoriasis Area and Severity Index and BSA—body surface area), the impact of psoriasis on the quality of life (evaluated using DLQI—Dermatological Life Quality Index), and anthropometric measurements—height and body weight (measured with a standardized weighing medical scale under fasting conditions). Body mass index (BMI) was calculated and evaluated according to formalized criteria (<18.5 kg/m^2^—underweight; 18.5–24.9 kg/m^2^—normal weight; 25.0–29.9 kg/m^2^—overweight; and ≥30.0 kg/m^2^—obesity) [18]. The following definition standards were used: type of psoriasis (I—age of onset <40.0 years old and II—age of onset ≥40.0 years old), PASI (<10.0 points—mild; 10.0–15.0 points—moderate; and >15.0 points—severe) [19], and DLQI score (0–1.0 points—no effect; 2.0–5.0 points—small effect; 6.0–10.0 points—moderate effect; 11.0–20.0 points—very large effect; and 21.0–30.0 points—extremely large effect) [20].

### 2.3. Statistical Analysis

For statistical analysis and data presentation, Statistica v. 12.0. (StatSoft, Inc., Tulsa, OK, USA, 2015) and Microsoft Office Excel (Microsoft Corporation, Redmond, WA, USA, 2018, version 16.16.27) were used. Qualitative feature analyses were conducted using the χ2 test with Pearson’s method. Independent variables that met the assumptions for parametric tests were analyzed using the *t*-Student test. Independent variables that did not meet the assumptions for parametric tests were analyzed using non-parametric tests, including the Mann–Whitney U test or the Kruskal–Wallis test. The relationship between quantitative variables was examined using Spearman’s rank correlation.

## 3. Results

The study cohort consisted of 147 patients diagnosed with psoriasis (mean age 45.51 ± 15.68 years, age range 18.0–83.0 years). Among these participants, 43 (29.25%) were female (mean age 48.91 ± 18.32 years, age range 18.0–83.0 years), and 104 (70.75%) were male (mean age 44.41 ± 14.32 years, age range 18.0–71.0 years). Psoriasis group characteristics are summarized in Table 1 and Table 2.

Type I psoriasis was predominantly more prevalent (76.2%) among patients, both men (78.85%) and women (69.77%), which corresponds with the young age of onset (mean 27.6 ± 15.4 y/o in general). More than half of the patients (64.38%) had nail psoriasis, and the incidences were comparable between men and women. On the other hand, psoriatic arthritis was less frequent (18.37% in general) and considerably more often reported in men (22.12%) than in women (9.30%).

In regards to psoriasis severity, men tended to have greater PASI and BSA values than women; however, the DLQIs were comparable for these groups.

In the context of BMI values, psoriasis patients in general had a strong predisposition towards being overweight (mean BMI 29.03 ± 5.14). There were no statistically significant discrepancies between BMI value and disease severity (defined by PASI, BSA, and DLQI values) between type I and type II psoriasis patients.

### 3.1. Analysis of Overweightness and Obesity Prevalences among Psoriatic Patients

**Figure 1:**
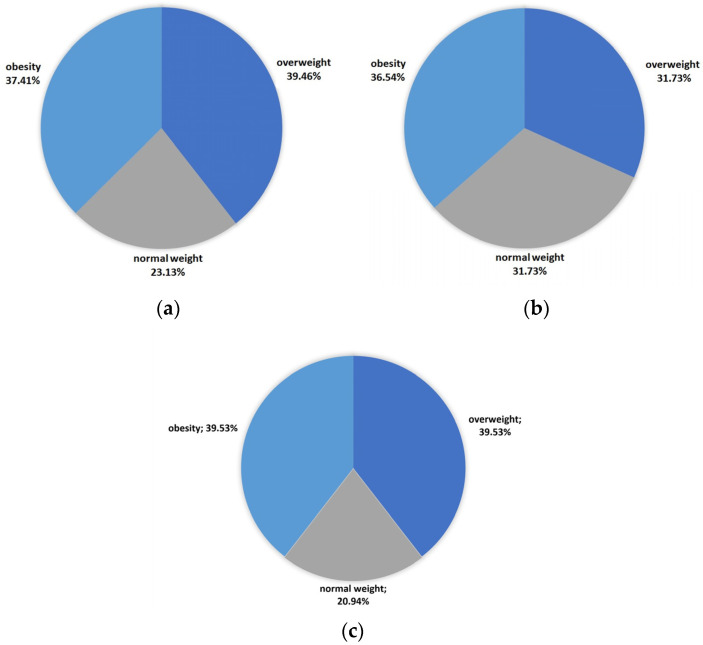
Analysis of the prevalences of normal weight, overweightness, and obesity in regards to the BMI value in the study group: (**a**) all patients; (**b**) men; and (**c**) women.

Figure 1 presents the evaluation of the BMI index in the study group. Overweightness (39.46%) and obesity (37.41%) were major phenomena in the psoriatic group. Merely 20% of psoriatic patients in general had a normal weight, whereas, about 80% of women and 70% of men had an abnormal body weight.

### 3.2. Analysis of the Role of Environmental Factors in Psoriatic Patients

A total of 83.56% of psoriatic patients, both men and women, declared they overlooked the importance of maintaining a balanced and nutritious diet. More than half of the patients (55.78%) were stated to be past or current smokers. The trend was more prominent in the women’s group (62.50%) than in the men’s (39.53%). Detailed analysis is provided in Table 3.

### 3.3. Analysis of the Body Mass Influence on Psoriasis Disease Course and Severity

**Figure 2:**
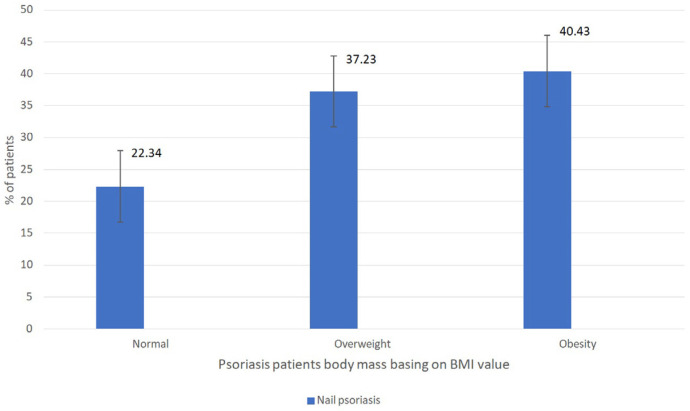
Analysis of body mass (in regards to the BMI value) among patients with nail psoriasis (*p* = 0.63).

**Figure 3:**
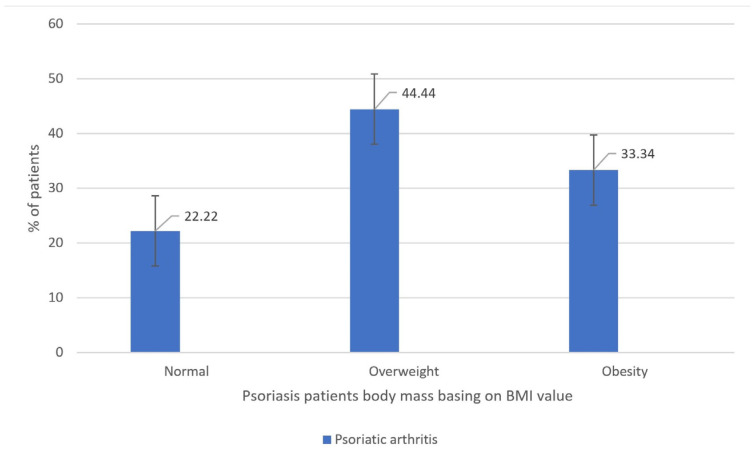
Analysis of body mass (in regards to the BMI value) among patients with psoriasis and psoriatic arthritis (*p* = 0.83).

In the context of both type I and type II psoriasis cohorts, no statistically significant correlations were found between the BMI index and various parameters, including the severity of psoriasis (as defined by PASI, BSA, and DLQI scores), incidence of psoriatic arthritis, presence of nail psoriasis, and cigarette smoking habits. Among overweight and obese patients, psoriasis nail involvement (77.66%) and psoriatic arthritis (77.77%) were more common in comparison to normal-weight patients, as depicted in Figure 2 and Figure 3.

**Figure 4:**
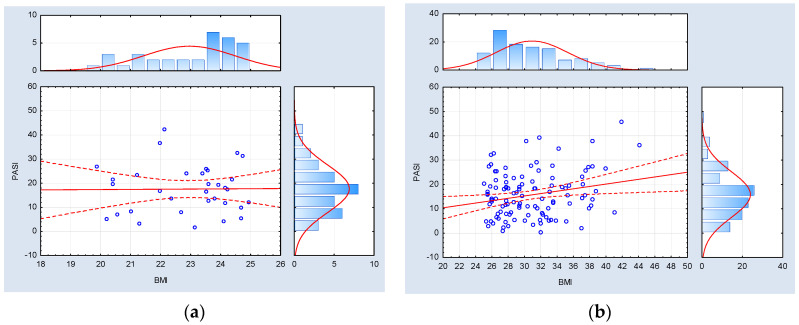
Correlation analysis between the BMI index (body mass index) and the PASI index (psoriasis area and severity index) among psoriatic patients with (**a**) normal weight (BMI < 25.00) (*n* = 34, Spearman R = −0.018342, and *p* = 0.917997) and (**b**) abnormal body weight (BMI ≥ 25.00) (*n* = 112, Spearman R = 0.23, and *p* = 0.016).

**Figure 5:**
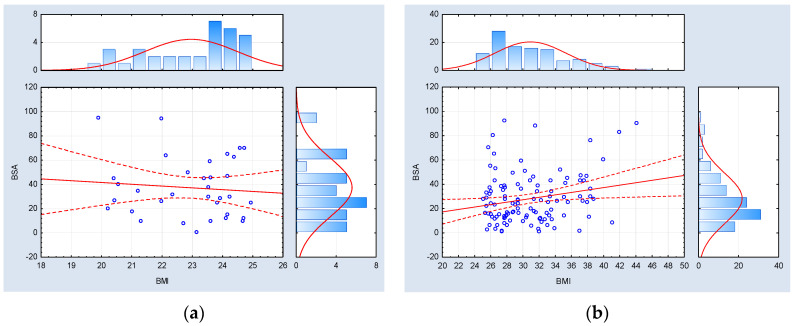
Correlation analysis between the BMI index (body mass index) and the BSA index (body surface area) among psoriatic patients with (**a**) normal weight (BMI < 25.00) (*n* = 34, Spearman R = −0.024086, and *p* = 0.892444) and (**b**) abnormal body weight (BMI ≥ 25.00) (*n* = 113, Spearman R = 0.21, and *p* = 0.023).

**Figure 6:**
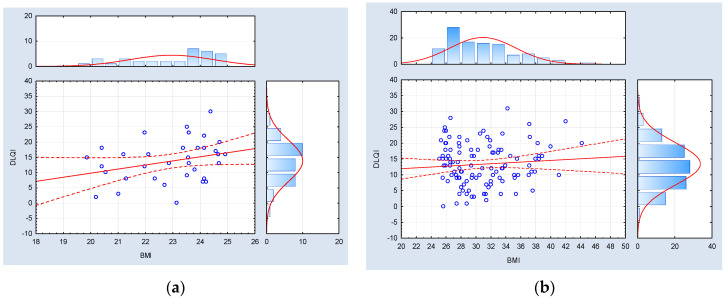
Correlation analysis between the BMI index (body mass index) and the DLQI index (dermatological life quality index) among psoriatic patients with (**a**) normal weight (BMI < 25.00) (*n* = 34, Spearman R = 0.309308, and *p* = 0.075070) and (**b**) abnormal body weight (BMI ≥ 25.00) (*n* = 112, Spearman R = 0.09, and *p* = 0.36).

Furthermore, in the cohort of psoriatic patients characterized by a normal BMI index, no correlations of statistical significance were identified between BMI metrics and the severity of psoriasis, as assessed by the PASI, BSA, and DLQI indexes. Conversely, within the subset of psoriatic patients with an abnormal body mass (inclusive of both overweight and obese individuals), statistically significant positive associations were observed between BMI values and the PASI and BSA indexes, whereas no such correlation was established with respect to the DLQI index. The data referred to above has been graphically represented in Figure 4, Figure 5 and Figure 6.

## 4. Discussion

Our study provided relevant evidence that overweightness and obesity constitute a major health burden among psoriatic patients by influencing the disease course and severity. Enhanced understanding of the phenomenon may directly translate into improving disease management and overall patient care.

The results of our study are consistent with published epidemiological studies considering the psoriasis disease characteristics, validating the representativeness of our study group. Comparing the large-scale cross-sectional cohort population-based study among psoriatic patients of Polish descent, early-onset psoriasis (type I) dominates (78.24%), along with the high incidence of nail psoriasis (63.61%) and moderate-to-severe disease manifestation (mean PASI 12.63) [21]. These data are also in line with worldwide studies carried out among populations of European [22] and Asian descent [23].

The prevalences of overweightness (39.46%) and obesity (37.41%) demonstrated in the study shows the strong predisposition of psoriatic patients for abnormal body mass. Furthermore, the association is strong, especially in the context of obesity, when compared to the latest reports on the prevalences of overweightness and obesity among the general population of Poland. (Stoś et al. reported, in the nationwide cross-sectional survey, 42.2% of overweightness and 16.4% of obesity among participants) [24]. Manifold studies also reported a high incidence of overweightness and obesity among psoriatic patients, regardless of ethnicity [25,26]. Moreover, obesity independently constitutes a risk factor associated with the onset of psoriasis [27,28].

Although we did not find any correlations between psoriasis types and BMI index values, some authors reported a higher prevalence of obesity among late-onset psoriasis subjects [29]. It is hypothesized that the genetic background (*HLA-C∗06:02* allele linked with early-onset psoriasis) may influence lower central adiposity and waist circumference, thus providing a cardiometabolic psoriasis biomarker [30,31,32].

Psoriatic involvement of the nails was observed in a substantial majority (64.38%) of cases, aligning closely with the findings reported by Brazzelli et al. (77% of psoriatic patients). However, in contrast to the authors’ observations of a significant association with psoriatic arthritis incidence (85.7%) [33], our study did not establish a similar correlation. Merely 22.12% of patients received a diagnosis of psoriatic arthritis. This may imply a pressing concern of potential underdiagnosis and underscores the imperative for early detection in clinical practice. Tłustochowicz et al. described in recent years a gradual rise in psoriatic arthritis detection, which confirms our observations [34].

It is noteworthy that among patients exhibiting both nail psoriasis and psoriatic arthritis, there were elevated prevalences of overweightness and obesity, which may suggest a comorbid nature underlying these conditions. To date, there are no studies evaluating the direct relationship between nail psoriasis and body mass. Nonetheless, it is possible that nail psoriasis might be indirectly linked to obesity, due to the elevated prevalence of psoriatic arthritis, a condition associated with both comorbidities [35]. The vast majority (77%) of subjects with psoriatic arthritis were overweight or obese. An elevated BMI index is linked to an increased susceptibility to psoriatic arthritis and its early onset [36,37,38]. Adipose tissue promotes the activation of inflammatory pathways through manifold adipokines (chiefly adipokine and leptin) and proinflammatory cytokines (such as TNF-alpha, IL-1, IL-6, IL-17, and IL-23), which share a chronic systemic inflammatory background with both psoriasis and psoriatic arthritis. Reversely, the decrease in functional ability associated with psoriatic arthritis leads to a reduction in physical activity, which in turn promotes weight gain and consecutive mechanical trauma to tissues [39,40]. This underscores the importance of body mass prevention and early treatment among patients with psoriasis and psoriatic arthritis.

Environmental factors and lifestyle changes significantly contribute to both the clinical presentation and the effective management of psoriasis. Despite the high prevalences of overweightness and obesity among psoriatic patients, 83.56% of subjects admitted to neglecting healthy dietary habits. Studies evaluating the impact of saturated fatty acids in mouse models with induced obesity and psoriasiform inflammation revealed them to serve as triggers of the exacerbation of skin lesions [41]. On the other hand, a very-low-calorie ketogenic diet, through antioxidant and anti-inflammatory effects and subsequent weight loss, significantly enabled better psoriasis disease control [42]. In comparison to the general population, a substantial percentage (55.78%) of psoriatic patients were declared to be past or current smokers, which contributes to a more severe course of the disease, as well as to the development of cardiovascular disease and a higher mortality rate [43,44,45,46,47]. Owczarczyk-Saczonek et al. noted a higher prevalence of tobacco addiction among Polish men with psoriasis [48]. Nonetheless, our findings revealed a noteworthy shift in smoking habits, with the majority of women (62.50%) having a history of past or current smoking. This trend could be indicative of changing societal norms and the impact of public health campaigns, which have historically been primarily directed towards men. Also, gender disparities in smoking addiction pose distinct challenges for women in achieving cessation. The presence of psoriasis, which is known to contribute to mood disorders and depression, may exacerbate this issue further [49].

The results of the correlation analysis revealed the significant impact of overweightness and obesity, as defined by the BMI index, on modifying the severity of psoriasis (as assessed by the PASI with a correlation coefficient of R = 0.23, *p* = 0.016; and BSA values with a correlation coefficient of R = 0.21, *p* = 0.023), particularly in contrast to patients with normal body mass. Bhole et al. further confirmed this association in a case-control study involving 448 psoriasis patients, demonstrating a significant relationship between obesity and elevated PASI scores (OR = 1.03; 95% CI, 1.01–1.05) [50]. Other researchers have also noted various degrees of correlation between psoriasis severity, ranging from weak to significant. A cross-sectional study involving 296 psoriatic patients of Latin American descent reported a minor positive association between PASI and BMI values (R = 0.0154, *p* = 0.01) [51]. Ahdout et al. described a notable positive correlation between the PASI and BMI among 65 psoriatic patients (R = 0.35, *p* = 0.004) [52]. Finally, Murray et al. performed an analysis including the results of 88 psoriasis patients showing a positive correlation between BMI and BSA indexes (R = 0.24, *p* = 0.02) [53]. The positive correlation between the overabundance of adipose tissue and the severity of psoriasis carries several adverse clinical implications for patients. These include a diminished response to treatment [12], leading to systemic therapeutic interventions that generate additional costs and potential pharmacological side effects. Moreover, it elevates the likelihood of concomitant cardiovascular diseases, which impacts both morbidity and mortality rates [54]. Severe psoriatic plaques can exacerbate mood disorders and impede physical activity, contributing to a reinforcing cycle of obesity among individuals with psoriasis.

Current therapeutic modalities for psoriasis encompass a spectrum of topical and systemic interventions, depending on the severity, localization, and extensiveness of the disease. Topical treatment is a first-line method reserved for mild cases and involves the application of highly potent glucocorticosteroids combined with vitamin D analogues, calcineurin inhibitors, and ditranol. For more severe cases, phototherapy (UVB, UVA, narrow band UVB 311 nm, and PUVA) and traditional systemic therapies, such as acitretin, methotrexate, and fumarates (apremilast, dimethyl fumarate), along with cyclosporine are available. However, the concomitance of obesity and psoriasis introduces therapeutic challenges. Limited topical pharmacotherapeutic options can be introduced among obese psoriatic patients, due to their greater skin surface area and greater pharmacoeconomic cost. Furthermore, there is an increased incidence of contraindications due to concurrent conditions, such as hyperlipidemia in the case of acitretin or hepatic steatosis for methotrexate. Also, the prevalence for adverse reactions escalates, for example by phototherapy-induced burns and polypharmacy, due to the treatment of concomitant diseases. On the other hand, the recent advancement in psoriasis treatment underscores the potential efficacy and safety of biologic treatments and small molecules, particularly in pediatric and geriatric populations. Biologic treatment encompasses a wide range of therapies, including tumor necrosis factor alpha inhibitors (infliximab, adalimumab, etanercept, and certolizumab pegol), an interleukin 12/23 inhibitor (ustekinumab), interleukin 17 inhibitors (secukinumab, ixekizumab, and brodalumab), and interleukin 23 inhibitors (risankizumab, guselkumab, and tildrakizumab). The latest research focuses on the implementation of small particles like JAK inhibitors (primarily tofacitinib and baricitinib) into a psoriasis therapeutic portfolio [55,56].

## 5. Limitations of the Study

On the other hand, our study has several limitations. Firstly, our findings should be confirmed through large cohort studies. Additionally, incorporating a control group for comparative analyses and calculating odds ratios (OR) would have strengthened the statistical rigor of the study. However, we managed to compare obtained data with relevant epidemiological published research. Furthermore, our study focused exclusively on adult individuals. Future investigations may benefit from including a pediatric population to explore early interventions for obesity prophylaxis in this patient demographic group. A more detailed examination of dietary habits, including the assessment of specific components, such as high-fat content, could have provided deeper insights into the relationship between diet and psoriasis. Addressing these limitations in future research may contribute to a more comprehensive understanding of the interplay between obesity and psoriasis.

## 6. Conclusions

Overweightness and obesity represent significant clinical determinants influencing the course of psoriasis. Prioritizing weight loss promotion and implementing early obesity prevention should be considered essential therapeutic interventions for psoriatic patients. This approach is crucial not only for enhancing disease management but also for preventing the onset of concomitant metabolic disorders.

## Figures and Tables

**Table 1:** Psoriasis group characteristics—clinical data.

Variable	General *n* = 147	Men *n* = 104	Women *n* = 43	*p*-Value (Difference between Men and Women)
Mean age (years ± SD)	45.5 ± 15.7	44.1 ± 14.3	48.9 ± 18.3	0.07
Mean age of chronic plaque psoriasis onset (years ± SD)	27.6 ± 15.4	27.6 ± 13.7	27.7 ± 18.9	0.39
Type of chronic plaque psoriasis (%)				0.24
I	112 (76.20%)	82 (78.85%)	30 (69.77%)	
II	35 (23.80%)	22 (21.15%)	13 (30.23%)	
Nail involvement (%)				0.90
present	94 (64.38%)	66 (64.08%)	28 (65.12%)	
absent	52 (35.62%)	37 (35.92%)	15 (34.88%)	
Psoriatic arthritis diagnosis (%)				0.07
present	27 (18.37%)	23 (22.12%)	4 (9.30%)	
absent	120 (81.63%)	81 (77.88%)	39 (90.70%)	

**Table 2:** Psoriasis group characteristics—disease severity and anthropometric measurements.

Variable	General *n* = 147	Men *n* = 104	Women *n* = 43	*p*-Value (Difference between Men and Women)	Type I Psoriasis *n* = 112	Type II Psoriasis *n* = 35	*p*-Value (Difference between Type I and Type II)
Mean PASI (points ± SD)	16.14 ± 9.49	17.59 ± 10.19	12.63 ± 6.36	0.007	15.97 ± 9.79	16.70 ± 8.54	0.47
Mean BSA (% ± SD)	30.27 ± 21.68	31.88 ± 23.87	26.39 ± 14.74	0.53	29.97 ± 22.09	31.21 ± 20.59	0.63
Mean DLQI (points ± SD)	13.05 ± 6.97	13.37 ± 7.41	12.28 ± 5.76	0.39	12.69 ± 7.21	14.24 ± 6.04	0.19
Mean BMI (points ± SD)	29.03 ± 5.14	28.96 ± 5.13	29.18 ± 5.21	0.71	28.91 ± 5.22	29.41 ± 4.94	0.46

Abbreviations: PASI—psoriasis area and severity index, SD—standard deviation, BSA—body surface area, DLQI—dermatological life quality index, BMI—body mass index.

**Table 3:** The role of environmental factors (diet and smoking) on psoriatic patients.

Variable	General *n* = 147	Men *n* = 104	Women *n* = 43	*p*-Value (Difference between Men and Women)
Diet				0.05
Healthy dietary habits (%)	24 (16.44%)	21 (20.39%)	3 (6.98%)	
No dietary habits (%)	122 (83.56%)	82 (79.61%)	40 (93.02%)	
Cigarette smoking				0.01
Current and past smokers (%)	82 (55.78%)	17 (39.53%)	65 (62.50%)	
Non-smokers (%)	65 (44.22%)	26 (60.47%)	39 (37.50%)	

## Data Availability

Data are contained within the article.
